# Optimal IRS Allocation and Relay Selection for mmWave Multi-Hop Communications for Vehicular Sensor Data Sharing

**DOI:** 10.3390/s26123837

**Published:** 2026-06-16

**Authors:** Xiaojun Yin, Xuyang Du, Xiaohan Wu, Xinming Zhang

**Affiliations:** School of Computer Science and Technology, University of Science and Technology of China, Hefei 230026, China; yxj66888@mail.ustc.edu.cn (X.Y.); duxuyang@mail.ustc.edu.cn (X.D.); xiaohanwu@mail.ustc.edu.cn (X.W.)

**Keywords:** cooperative perception, IRS, millimeter-wave communication, relay selection, sensor data sharing, vehicular networks

## Abstract

Modern connected and automated vehicles are equipped with various onboard sensors, which continuously generate high-rate perception data. The reliable and timely sharing of such sensor data among neighboring vehicles requires high-capacity and low-latency vehicle-to-vehicle (V2V) communications. Millimeter-wave (mmWave) technology is a promising solution for supporting such high-rate transmission. However, mmWave V2V communication may be severely affected by non-line-of-sight (NLOS) blockage caused by limited transmission range, roadside obstacles, and moving vehicles. Relay forwarding can improve communication reliability and extend transmission distance, while intelligent reflecting surfaces (IRSs) can construct virtual line-of-sight (LOS) links to mitigate NLOS blockage. In this paper, we propose deploying IRSs on urban roadsides to improve mmWave multi-hop V2V communication for vehicular sensor-data sharing by integrating IRS-assisted link selection into multi-hop relay forwarding. However, IRS deployment introduces new challenges in relay selection and directional transmission coordination under interference. To address these challenges, we propose an IRS allocation and relay selection (IARS) scheme for IRS-assisted multi-hop V2V communication. The proposed scheme is based on a transmission evaluation function that jointly considers inter-vehicle distance, link quality, and concurrent transmissions. Simulation results show that the proposed IARS scheme can effectively improve communication reliability and reduce multi-hop delay, thereby supporting reliable and timely sensor-data sharing in urban vehicular networks.

## 1. Introduction

With the rapid development of connected and automated vehicles (CAVs), modern vehicles are increasingly equipped with heterogeneous onboard sensors, such as cameras, light detection and ranging (LiDAR), radar, and inertial sensors, to perceive the surrounding traffic environment and support autonomous driving, cooperative driving, and driving safety applications [1,2]. These sensors continuously generate rich perception data, including high-resolution images, point clouds, and object-level environmental information. For example, automotive cameras and LiDAR sensors may generate data streams at hundreds of Mbps, especially when raw or lightly compressed perception data needs to be exchanged for cooperative perception and sensor-data sharing [3,4]. By sharing sensor data among neighboring vehicles, the sensing range of an individual vehicle can be extended beyond its own field of view, thereby improving the awareness of occluded vehicles, pedestrians, intersections, and other safety-critical objects. Therefore, reliable and low-latency transmission of high-rate sensor data has become an important requirement for future vehicular networks.

As a key enabler for high-data-rate vehicular communications in 5G/B5G networks, millimeter-wave (mmWave) technology has been introduced into vehicular networks to support real-time sensor-data sharing and cooperative perception, due to its potential to provide high data rates and low latency [4,5,6]. However, high-frequency mmWave signals may suffer from severe fading and large penetration loss, which significantly limit their transmission range [7]. Through beamforming and directional transmission technologies, higher antenna gains can be obtained in the transmission direction, compensating for the large propagation losses of mmWave signals [5,8]. Nevertheless, mmWave signals still suffer from severe penetration loss and have difficulty penetrating obstacles. In urban environments, high-rise buildings, roadside objects, and moving vehicles may block mmWave links, making mmWave communication highly dependent on line-of-sight (LOS) paths [9,10]. Roadside assistance and coordinated scheduling can improve transmission opportunities in dense urban environments [6,11]. However, relying on dense infrastructure support would increase deployment cost and coordination complexity, which may restrict the large-scale deployment of sensor-data sharing services.

Therefore, a practical way to overcome blockage and improve LOS coverage with limited infrastructure support is to select vehicles as relay nodes for multi-hop vehicle-to-vehicle (V2V) communication [12,13]. Multi-hop V2V communication can extend the transmission range of sensor data and improve network coverage, but numerous hops may also lead to increased latency. This is particularly undesirable for perception-driven vehicular applications, where outdated camera or LiDAR data may reduce the effectiveness of cooperative perception and even affect driving safety. In addition, the relay forwarding performance of multi-hop V2V communication may be affected by traffic flow and network topology. In areas where vehicles are densely distributed, such as intersections with peak traffic, direct V2V links between vehicles may be blocked by other vehicles, and the distance of single-hop transmission may be severely limited [10]. In sparsely populated areas, such as streets at midnight, inter-vehicle connectivity may be limited by the small number of neighboring vehicles. As a result, the number of dead nodes increases, and multi-hop V2V communications may suffer from high outage probabilities, which further weakens the reliability of sensor-data dissemination.

**Figure 1:**
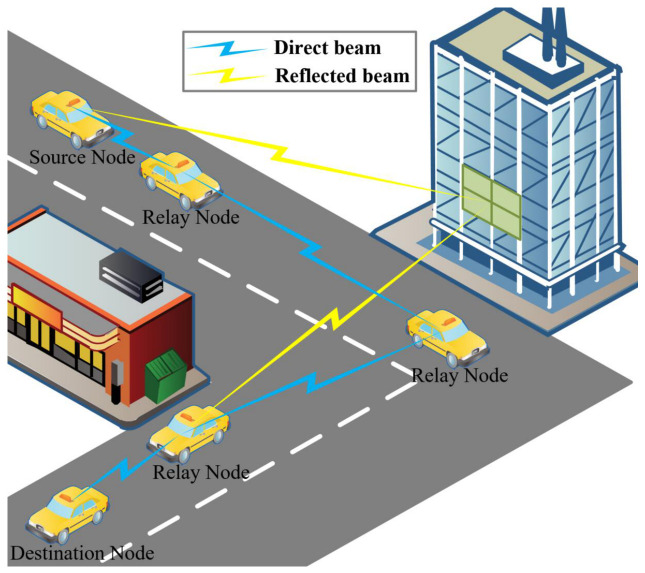
Deploying IRS to assist multi-hop V2V communications.

Establishing virtual LOS links through intelligent reflecting surface (IRS) reflections has become a promising approach for extending transmission distance and mitigating non-line-of-sight (NLOS) blockage. Recent surveys have highlighted IRS technology as an important means of creating a programmable wireless environment for vehicular communications [14]. An IRS consists of numerous configurable low-cost passive reflective elements, which can independently change the amplitude and phase of the reflected signals. Therefore, by optimizing the reflection coefficient of each element, the reflected signals from all elements can be superimposed and strengthened at a specific position, thereby improving the signal strength received by the receiver. By deploying IRSs at suitable locations and optimizing their element configurations, mmWave signals can be transmitted through reflected links to extend transmission distance and bypass obstacles. In this way, IRS-assisted mmWave transmission can extend the coverage of single-hop V2V links and reduce the probability of forwarding failures, which is beneficial for delay-sensitive sensor-data sharing in urban vehicular networks. The ability of IRSs to extend single-hop transmission distance motivates us to introduce IRS assistance into mmWave multi-hop V2V communication, as shown in Figure 1.

In summary, the problem addressed in this paper is how to support reliable and low-latency transmission of high-rate vehicular sensor data in urban mmWave multi-hop V2V networks under blockage, topology dynamics, and interference constraints. Although relay forwarding can extend the communication range, excessive relay hops may increase end-to-end delay. Meanwhile, IRS-assisted reflection links can mitigate NLOS blockage and improve single-hop transmission opportunities, but IRS resources are limited and their reflected signals may introduce additional interference. Therefore, for each time slot, it is necessary to determine which transmitters should be assisted by IRSs, whether a direct or IRS-assisted link should be used, and which vehicle should be selected as the next-hop relay. The objective is to improve the forwarding progress and link reliability of concurrent transmissions, thereby reducing the overall multi-hop transmission delay. To address this problem, we design an IRS allocation and relay selection scheme, called IARS, for IRS-assisted mmWave multi-hop V2V communication to reduce the overall multi-hop latency while ensuring transmission reliability. The contributions of this paper are summarized as follows:

- We propose to deploy IRSs in vehicular networks to assist mmWave multi-hop V2V communication, aiming to support reliable and low-latency transmission of high-rate vehicular sensor data.
- We integrate IRS-assisted link selection into relay-based multi-hop V2V communication. These heterogeneous links are then jointly considered in the relay selection and scheduling process to construct an efficient end-to-end multi-hop forwarding path.
- Taking the influence of interference into account, we design an IRS allocation algorithm and a relay selection algorithm for IRS-assisted mmWave multi-hop V2V communication.
- We conduct extensive SUMO-based simulations under different transmit powers, vehicle densities, IRS resource configurations, and grid sizes. The results demonstrate the effectiveness and scalability of the proposed IARS scheme in improving delivery reliability and multi-hop delay performance.

## 2. Related Work

Recent studies on mmWave vehicular networking have mainly focused on directional link establishment, scheduling, and blockage characterization. Tan et al. [5] surveyed beam alignment techniques for mmWave vehicle-to-everything (V2X) communications and showed that rapid mobility, narrow beams, and imperfect sensing information make reliable link establishment a major bottleneck. He et al. [6] formulated a Sub-6 GHz-assisted mmWave V2X scheduling problem with explicit inter-link conflicts, data importance, and data freshness, and further studied the scheduling-phase design in [11]. Xie et al. [15] investigated learning-assisted user scheduling and beamforming for mmWave vehicular networks without relying on explicit channel state information (CSI). Meanwhile, recent channel measurements further quantified the pronounced impact of vehicular blockage on mmWave propagation in V2V scenarios [10,16]. These studies provide important insights into directional scheduling and blockage-aware design, but they do not address multi-hop relay selection with explicit IRS resource competition.

Multi-hop relaying remains a practical way to extend mmWave V2V coverage when direct LOS links are unavailable. Recent studies have shown that learning-based beam and relay coordination can effectively mitigate blockage and improve vehicular communication performance. For example, Ju et al. [17] proposed a deep reinforcement learning-based joint beam allocation and relay selection scheme for mmWave vehicular networks. However, such relay-oriented studies mainly optimize beam/relay decisions in conventional vehicular settings and do not consider the additional coupling introduced by roadside IRS deployment, where a transmitter may compete with others for limited reflective resources and may switch between direct and reflected paths when selecting the next hop.

In parallel, RIS/IRS-enabled vehicular communications have attracted increasing attention. Recent surveys summarized the architectural variants, channel acquisition methods, and optimization opportunities of RIS-enabled vehicular systems [14]. Badheka et al. [18] further refined the communication-oriented modeling of intelligent reflecting surfaces by bridging physical electromagnetic formulations and system-level models. For practical roadside deployment, Huang et al. [8] proposed efficient channel estimation and low-complexity beamforming for IRS-aided vehicular communication. Shi et al. [19] further developed a three-dimensional beam-domain channel modeling and analysis framework for RIS-empowered V2V communications. These studies strengthen the modeling and signal-processing foundation of RIS-assisted vehicular networks, but they mainly focus on single-link performance, channel estimation, or channel characterization rather than end-to-end multi-hop forwarding.

A growing body of work has begun to investigate how RIS assistance interacts with relay-based transmission. Zhou et al. [12] compared AF relays and passive RIS for V2V reliability enhancement, clarifying the performance tradeoff between active forwarding and passive reflection. Ji et al. [20] studied vehicular communication based on RIS-assisted sources and compared RIS-relay with energy-harvesting relay architectures, while Ji et al. [21] further developed a cooperative transmission algorithm for RIS-assisted intelligent transportation systems under aggregated interference. More recently, Guan et al. [13] considered IRS-assisted multi-hop vehicular collaborative communication by jointly optimizing link selection and IRS reflection coefficients. Their study is closely related to our work because both aim to reduce the delay of IRS-assisted multi-hop vehicular communication. Nevertheless, our work differs from Guan et al. [13] in three key aspects. First, Guan et al. mainly focus on link selection and reflection-coefficient optimization, whereas this paper further treats IRS assistance as a limited network resource that must be allocated among competing transmitters. Second, the proposed scheme explicitly models directional concurrent transmissions and checks interference conflicts among selected direct and reflected links, which is essential for mmWave V2V scheduling in dense urban scenarios. Third, our relay selection is coupled with IRS allocation: reflection links are first prioritized according to their improvement over direct transmission and the service capacity of each IRS, and the remaining transmitters are then scheduled through a conflict-aware relay selection graph. Although DRL-based relay selection has also shown promising performance in mmWave vehicular networks [17], such methods usually require task-specific state, action, and reward designs as well as sufficient training under the target scenario. Therefore, an interference-aware and lightweight IRS allocation and relay selection framework tailored to urban mmWave multi-hop V2V communication is still needed.

As summarized in Table 1, existing studies have improved mmWave V2X communication from different aspects, including beam alignment, link scheduling, blockage modeling, relay transmission, and IRS/RIS-assisted link enhancement. However, most of them focus on either link-level optimization or single-hop IRS assistance, while the coupling among IRS allocation, relay selection, and concurrent multi-hop scheduling remains insufficiently addressed. To fill this gap, this paper proposes an IRS-assisted relay scheduling scheme for urban mmWave V2V networks. The proposed scheme evaluates both direct and IRS-assisted links, allocates limited IRS resources to suitable transmitters, and selects relay links by jointly considering link quality, forwarding progress, and interference. In this way, the proposed method bridges IRS-assisted link enhancement and multi-hop relay forwarding, thereby improving the reliability and efficiency of mmWave V2V communication under urban blockage.

## 3. System Model and Problem Formalization

### 3.1. System Model

#### 3.1.1. Network Model

**Figure 2:**
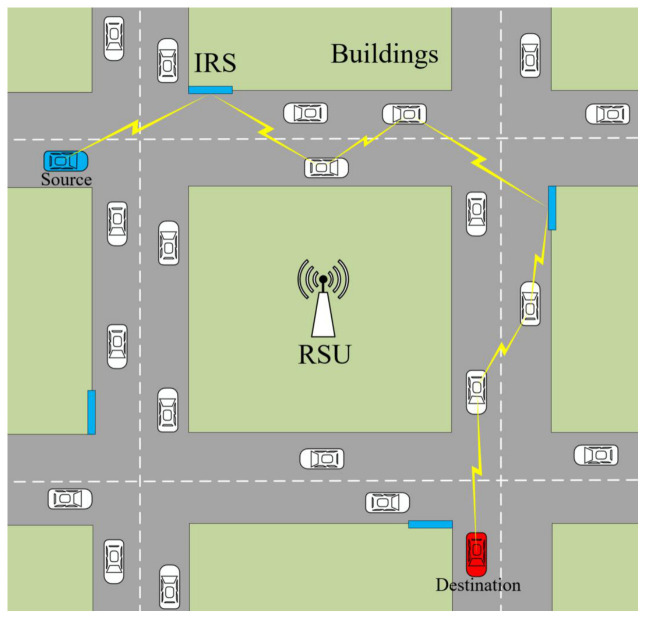
IRS-assisted urban mmWave multi-hop V2V communication network model.

We consider an mmWave multi-hop V2V communication network model in an urban scenario, as shown in Figure 2. Streets in the urban scenario have a typical grid topology, with horizontal and vertical roads perpendicular to each other at intersections. Some vehicles in the network are randomly selected as source nodes, each of which has data packets to transmit to its corresponding destination node. Without loss of generality, we consider the same packet length.

In the urban scenario, the transmission of mmWave signals may be blocked by obstacles. Buildings between streets, as well as vehicles traveling in the streets, can both act as obstacles to block the transmission of mmWave signals. For simplicity, when considering vehicles as obstacles, we assume that they are approximately the same size. Therefore, if the distance between a source node and the corresponding destination is sufficiently far, or the direct link is blocked by obstacles, the transmission of data packets needs to be completed through multi-hop relay forwarding.

Vehicles in the network are equipped with both mmWave and low-frequency dedicated short-range communication (DSRC) communication devices. Data are transmitted over mmWave V2V channels, while DSRC channels are used to broadcast GPS-based location and mobility information for neighbor discovery and beam alignment. IRSs are deployed on buildings near intersections to assist in wireless communication between vehicles.

Since IRSs are generally not equipped with computing components, infrastructure is required to perform configuration optimization and transmit control signals to them through wired links. In our network model, a roadside unit (RSU) is deployed to control the IRSs and provide full network coverage through DSRC channels. The RSU mainly performs the following two tasks. First, it collects vehicle information, performs IRS resource allocation and relay selection, and broadcasts control information through DSRC channels. Second, it optimizes IRS configurations and sends control signals to the IRSs. Although the coverage of mmWave vehicle-to-infrastructure (V2I) channels is limited, it is also a feasible solution to use RSUs as static relays to forward data packets when vehicles are sparsely distributed, but this is beyond the scope of this paper. In our multi-hop V2V communication network model, RSUs only send and receive control signals and do not participate in multi-hop data transmission.

#### 3.1.2. Time Slot Structure

**Figure 3:**
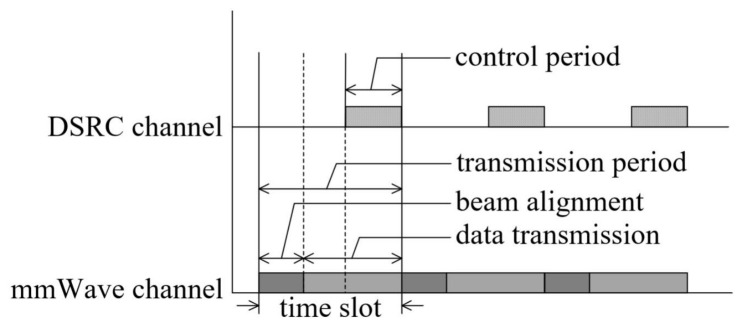
The structure of the time slot. Dark blocks represent the beam alignment stage, and light blocks represent the transmission stage.

To make IRS resource allocation and relay-node selection decisions, the RSU collects vehicle mobility information through DSRC channels, obtains inter-vehicle distance and obstacle information, and estimates link quality. After calculating the transmission schedule, the RSU also needs to broadcast control information through DSRC. In a high-speed vehicular network, vehicle mobility information is only valid for a short period of time. Therefore, the collection of mobility information needs to be performed periodically in each time slot. As shown in Figure 3, we divide each time slot into a control period and a transmission period.

During the control period, the RSU completes information collection, scheduling decisions, and control-information broadcasting. Specifically, vehicles first send their mobility information and packet-holding status to the RSU through DSRC channels. The RSU then makes scheduling decisions according to the IRS allocation and relay selection scheme proposed in the next section. Finally, through DSRC broadcast, the RSU sends control packets to vehicles.

A transmission period is divided into two stages: mmWave beam alignment and data transmissions. For beam alignment of mmWave signals, vehicle mobility information can be used to accelerate the beam scanning process [5]. In the transmission period, after a vehicle receives a control signal sent by the RSU, if it is scheduled to participate in data transmission, it first completes the beam alignment, and then sends or receives the corresponding data packet.

The control information transmission in the control period is done through DSRC channels, while the data transmission in the transmission period is only done through mmWave channels. Therefore, RSUs can collect vehicle information, calculate scheduling decisions, and send control information through DSRC channels when data transmission is carried out through mmWave channels. In other words, the control period of the next time slot can partially overlap with the transmission period of the previous time slot. When the mmWave signal is transmitted in the transmission period of the previous time slot, information collection, scheduling decision calculation and control signal transmission are performed on the DSRC channel in advance.

Therefore, the proportion of effective transmission time in each time slot is increased, while the overall transmission delay is reduced. Let $T_{ctrl}$ and $T_{tran}$ denote the length of a control period and that of a transmission period, respectively; through this optimization method, as shown in Figure 3, we can reduce the length of each time slot from $T=T_{ctrl}+T_{tran}$ to $T=\text{max}(T_{ctrl},T_{tran})$. For convenience of analysis, and because control packets only carry compact mobility updates and scheduling information, we assume that $T_{ctrl}<T_{tran}$ and $T=T_{tran}$.

The vehicle topology and channel conditions are assumed to be quasi-static within one time slot, while they may change across different time slots. This assumption is consistent with the slot-based scheduling framework, where control information is periodically updated. Vehicles operate in a half-duplex mode in each time slot, and a vehicle can act as at most one transmitter or one receiver in the same slot. For a given transmitter-receiver pair, only one transmission mode is selected in a time slot, namely either the direct mmWave link or one IRS-assisted reflection link. The ideal sector antenna model and the adopted path-loss model are used to estimate directional mmWave link quality and interference relationships. These assumptions make the proposed problem formulation tractable while preserving the key characteristics of IRS-assisted mmWave multi-hop V2V communication.

Although IRS phase/mode reconfiguration requires a finite hardware switching time in practical prototypes, we assume that the IRS configuration is determined before the corresponding mmWave data transmission starts and remains unchanged during the data transmission period. The IRS switching delay is therefore regarded as part of the control and guard interval between two consecutive transmission periods. This assumption requires the control period to be sufficiently larger than the IRS hardware switching time. For IRS prototypes with slower switching circuits, the control period can be enlarged, or the IRS configuration update frequency can be reduced accordingly. Thus, the proposed scheme does not rely on symbol-level instantaneous IRS reconfiguration.

#### 3.1.3. Antenna Pattern

**Figure 4:**
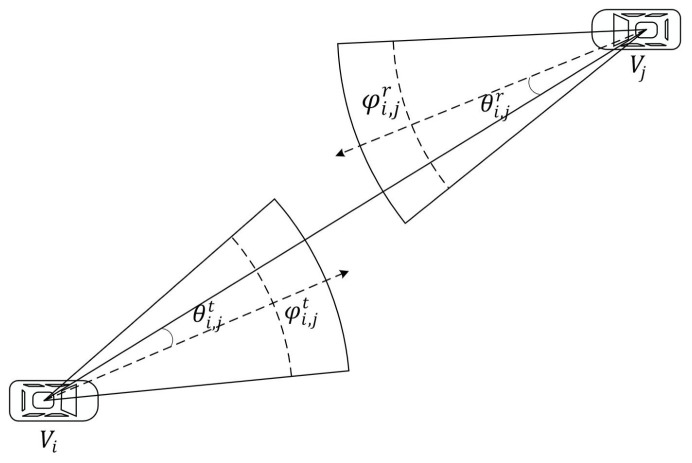
Ideal antenna sector model in the horizontal plane.

In mmWave V2V communication scenarios, since mmWave signals have very short wavelengths, large-scale antenna arrays with smaller dimensions can be integrated into communication devices to obtain a higher antenna gain in the transmission direction through beamforming and directional transmission technologies. We make a similar assumption in this paper: each vehicle is equipped with an mmWave antenna array and employs directional beamforming to enhance mmWave signal propagation. To maximize the directional gain of the mmWave antenna, beam alignment is performed at both the transmitting and the receiving vehicles. The directional antenna pattern generated by beam alignment is approximated as an ideal sector model in the horizontal plane, as shown in Figure 4.

Specifically, $l_{i,j}$ denotes the link between a transmitting vehicle $v_{i}$ and a receiving vehicle $v_{j}$. The antenna directivity gains $g_{k,i,j},\;k\in \left\{t,r\right\}$ of the transmitting vehicle and the receiving vehicle over link $l_{i,j}$ after beam alignment are as follows:

(1) $$g_{k,i,j}=\left\{\begin{array}{cc} \frac{2\pi -\left(2\pi -\phi_{i,j}^{k}\right)g_{0}}{\phi_{i,j}^{k}}, & \left|\theta_{i,j}^{k}\right|\leq \phi_{i,j}^{k}\\ g_{0}, & otherwise \end{array}\right.$$

where $0<g_{0}\ll 1$ is a constant representing the non-negligible side lobe gain. $g_{t,i,j}$ and $g_{r,i,j}$ are the transmit antenna gain and the receive antenna gain of link $l_{i,j}$, respectively. As shown in Figure 4, $\phi_{i,j}^{t}$ and $\phi_{i,j}^{r}$ represent the half-power beamwidths of the transmit antenna and the receive antenna, respectively. $\theta_{i,j}^{t}$ and $\theta_{i,j}^{r}$ represent the alignment error angles of the transmitter and receiver, respectively.

#### 3.1.4. Channel Model

MmWave signals suffer from a large path loss, atmospheric absorption and penetration loss, and are susceptible to blockage by obstacles. Recent measurements also continue to confirm the pronounced sensitivity of vehicular mmWave links to dynamic blockage [10]. To approximate the propagation loss of mmWave signals, we adopt a standard logarithmic distance path loss model for 60 GHz mmWave signals proposed in [22]. In this model, in addition to propagation distance and atmospheric absorption, the blocking effect of vehicle obstacles on mmWave signals is also considered. According to this model, the propagation loss ${Loss}_{i,j}$ between transmitter $v_{i}$ and receiver $v_{j}$ is expressed in decibels as follows:

(2) $${Loss}_{i,j}=10\delta_{i,j}{\text{log}}_{10}\left(d_{i,j}\right)+\eta_{i,j}+15d_{i,j}/1000$$

where $d_{i,j}$ is the Euclidean distance between transmitter $v_{i}$ and receiver $v_{j}$, in meters. $\delta_{i,j}$ is the path loss exponent and $\eta_{i,j}$ represents the effect of blocking on signals, and the values of these two parameters are determined by the number of obstacles between transmitter $v_{i}$ and receiver $v_{j}$. The last term represents the atmospheric absorption of 15 dB/km at 60 GHz. Recent measurement-based studies on mmWave vehicular propagation with vehicle obstructions further confirmed the strong dependence of channel attenuation on blockage conditions [16]. Accordingly, we retain the obstacle-dependent parameterization of $\delta_{i,j}$ and $\eta_{i,j}$ in our abstraction, so that more severely blocked links experience larger propagation loss. Let $p_{0}$ denote the transmit power of $v_{i}$. The received power $p_{i,j}$ over the direct link can then be obtained as follows:

(3) $$p_{i,j}=p_{0}g_{t,i,j}g_{l,i,j}g_{r,i,j}$$

where $g_{t,i,j}$ and $g_{r,i,j}$ are the transmitter and the receiver antenna gains, respectively. $g_{l,i,j}=10^{-\frac{{Loss}_{i,j}}{10}}$ is a linear form of the propagation loss.

Based on measurement-based path-loss characterization and communication-oriented RIS modeling, Tang et al. [23] and Yu et al. [24] studied practical modeling and configuration methods for intelligent reflecting surfaces. Motivated by the low-overhead discrete-control idea in [24], we adopt a tile-level transmission-mode abstraction for IRS configuration. Specifically, the whole reflective surface is divided into *N* tiles, and the configuration of each tile is selected from *M* optional transmission modes, where each transmission mode depends on its optimal incident angle $\Psi_{t}^{*}$ and reflection angle $\Psi_{r}^{*}$. The response function of the m-th transmission mode of the n-th tile of IRSs can be expressed as $G_{s,n,m}\left(\Psi_{t},\Psi_{r}\right)$, which represents the reflection gain at a certain incident angle $\Psi_{t}$ and reflection angle $\Psi_{r}$. When the actual incident angle and reflection angle are, respectively, equal to the optimal incident angle and reflection angle of the transmission mode, that is, $\Psi_{t}=\Psi_{t}^{*}$, $\Psi_{r}=\Psi_{r}^{*}$, the response function obtains the maximum value, $|G_{s,n,m}\left(\Psi_{t}^{*},\Psi_{r}^{*}\right)|=\frac{4\pi L_{tile}^{2}}{\lambda^{2}}$, where $L_{tile}$ is the side length of a tile. In this way, the IRS configuration is to select the transmission mode for all tiles, and then the superposition of these response functions forms the configuration of the whole IRS, expressed as $G_{s}=\sum_{n}^{N}\sum_{m}^{M}\beta_{s,n,m}G_{s,n,m}$, where $\beta_{s,n,m}$ is an element of the IRS configuration matrix β, and $\beta_{s,n,m}=1$ indicates that the n-th tile of IRSs selects the m-th transmission mode. Then, we can obtain the propagation loss ${Loss}_{i,j,s}$ between transmitter $v_{i}$ and receiver $v_{j}$ reflected by IRSs as follows [23]:

(4) $${Loss}_{i,j,s}={Loss}_{i,s}{\left|G_{i,j,s}\right|}^{2}{Loss}_{s,j}$$

where ${Loss}_{i,s}$ and ${Loss}_{s,j}$ are the propagation losses of the incident link and the reflection link, respectively, which can be calculated by (2). ${\left|G_{i,j,s}\right|}^{2}$ is the response gain of the reflection link for transmitter $v_{i}$, receiver $v_{j}$ and IRSs calculated based on the transmission mode. We can obtain the received power $p_{i,j,s}$ from the reflection link of IRSs as follows:

(5) $$p_{i,j,s}=p_{0}g_{t,i,s}g_{l,i,j,s}g_{r,s,j}$$

where, similarly, $g_{t,i,s}$ and $g_{r,s,j}$ are the antenna gains of transmitter $v_{i}$ and receiver $v_{j}$, respectively. $g_{l,i,j,s}$ is the propagation loss of the reflection link in a linear form.

For convenience of representation, we use s=0 to represent the case of direct links, that is, $p_{i,j,0}=p_{i,j}$. Based on the above calculations, we can estimate the quality of direct and reflection links. The signal-to-interference-and-noise ratio ${SINR}_{i,j,s}$ of a direct (s=0) or a reflection (s≠0) link can be obtained as follows:

(6) $${SINR}_{i,j,s}=\frac{p_{i,j,s}}{\sum_{x\neq i}\sum_{z\in S}p_{x,j,z}+N_{0}B}$$

where $p_{x,j,z}$ is the interference from a direct link (z=0) or a reflection link (z≠0) of another transmitter $v_{x}$. $N_{0}$ is the spectral power of the Gaussian noise and *B* is the bandwidth. In this way, we can obtain the data rate of a direct link or a reflection link, as $R_{i,j,s}=B\;{\text{log}}_{2}\left(1+SINR_{i,j,s}\right)$

### 3.2. Problem Formalization

Limited by the directional narrow beam of mmWave, the received power in the side lobe direction is negligible, and two vehicles can only choose one link for communication. Let i,j∈[1,N] represent the index of vehicles, s∈[0,S] represent the index of IRS. Specifically, for convenience, we use s=0 to denote the case of direct links.

The triple $\left(src_{d},des_{d},d\right)$ represents a transfer task sending data packet *d* from $v_{src_{d}}$ to $v_{des_{d}}$. Let $Q^{\tau }$ denote the holding packet information in time slot τ, where $Q_{i,d}^{\tau }=1$ indicates that $v_{i}$ holds packet *d* to be forwarded in time slot τ. Initially, for each transfer task sending packet *d* from $v_{src_{d}}$, let $Q_{src_{d},d}^{0}=1$, otherwise $Q_{i,d}^{0}=0$. When each packet reaches its destination, i.e., $Q_{des_{d},d}^{\tau }=1$ for every packet *d*, all transmissions are completed.

Before packet *d* reaches $v_{des_{d}}$, that is, $Q_{des_{d},d}^{\tau }=0$, the delay of packet *d* increases by $T_{\tau }$ in each time slot, where $T_{\tau }$ is the length of time slot τ. After $Q_{des_{d},d}^{\tau }=1$, the delay of packet *d* is no longer increased. Therefore, the overall delay can be expressed as $\sum_{d}\sum_{\tau }\left(1-Q_{des_{d},d}^{\tau }\right)T_{\tau }$. The direct goal of the IRS allocation and relay selection problem is to minimize the overall delay of multi-hop transmissions, that is, $\min_{\alpha }\sum_{d}\sum_{\tau }\left(1-Q_{des_{d},d}^{\tau }\right)T_{\tau }$. However, the overall optimization problem defined in this way is unsolvable, because new transmission tasks may appear at any time, the future state is uncertain, and the optimal decision based on the current situation may fail in the future. Therefore, we perform optimal relay selection for each time slot. Let $\alpha_{i,j,s,d}^{\tau }=1$ indicate that in time slot τ, $v_{i}$ sends data packet *d* to $v_{j}$ through the reflection link of IRSs (s≠0) or the direct link (s=0). $Dis_{i,des_{d}}^{\tau }$ and $Dis_{j,des_{d}}^{\tau }$ are the distances of $v_{i}$ and $v_{j}$ to the destination of data packet *d*, respectively. Our optimization goal is to maximize the total forwarding progress with the shortest possible delay in each slot, and the optimization problem is formulated as follows:

(7) $$\underset{\alpha^{\tau }}{\text{max}}\sum_{i}\alpha_{i,j,d,s}^{\tau }\left(Dis_{j,des_{d}}^{\tau }-Dis_{i,des_{d}}^{\tau }\right)/T_{\tau },$$

(8) $$\begin{array}{cc} s.t. & \alpha_{i,j,s,d}^{\tau }\in \left\{0,1\right\},\forall i,j\in \left[1,N\right],\forall s\in \left[0,S\right], \end{array}$$

(9) $$\begin{array}{c} \sum_{i,s,d}\alpha_{i,k,s,d}^{\tau }+\sum_{j,s,d}\alpha_{k,j,s,d}^{\tau }\leq 1,\forall k\in \left[1,N\right], \end{array}$$

(10) $$\begin{array}{c} \sum_{s,d}\alpha_{i,j,s,d}^{\tau }\leq 1,\forall i,j\in \left[1,N\right], \end{array}$$

(11) $$\begin{array}{c} Q_{k,d}^{\tau +1}=Q_{k,d}^{\tau }+\sum_{i,s}\alpha_{i,k,s,d}^{\tau }Q_{i,d}^{\tau }-\sum_{j,s}\alpha_{k,j,s,d}^{\tau }Q_{k,d}^{\tau }, \end{array}$$

(12) $$\begin{array}{c} \frac{DL_{d}}{R_{i,j,s,d}}<\left(T_{\tau }-T_{0}\right),\forall \alpha_{i,j,s,d}^{\tau }=1. \end{array}$$

where (8) and (9) constrain that in a time slot, a vehicle can only act as one of the transmitter or receiver to complete the transmission of a data packet through a link. Equation (10) indicates that a transmission pair can transmit at most one data packet over one link in each time slot. Equation (11) indicates that the relay forwarding of data packets changes the status of data packets held by vehicles. Equation (12) indicates the time delay limit, where $T_{0}$ is the beam alignment time, $DL_{d}$ is the length of data packet *d*, and the data rate $R_{i,j,s,d}$ of each scheduled transmission must ensure that the transmission can be completed within the time slot. Due to the nonlinearity of constraints, the optimization problem is a nonlinear optimization problem. The binary selection variables in Equations (8)–(12) indicate that different transmission modes are selected in an exclusive manner for each hop. This formulation reflects the practical constraint that a hop can be served by either a direct V2V link or an IRS-assisted reflected link, rather than by both simultaneously. Thus, the optimization problem is essentially a joint relay-selection and IRS-resource-allocation problem.The proposed model does not involve physical-layer signal combining, IRS-aided amplify-and-forward relaying, or cascaded relay channels. The integration of IRS-assisted transmission and relay forwarding is achieved at the network level: an end-to-end multi-hop path may contain both direct V2V hops and IRS-assisted V2V hops, and the proposed IARS scheme jointly selects relays and allocates IRS resources to improve path reliability and reduce forwarding delay.

It should be noted that the above formulation is derived from the original long-term delay minimization problem. Directly solving the long-term problem is impractical in dynamic vehicular networks, because vehicle positions, blockage conditions, packet arrivals, and interference relationships may change from slot to slot. Moreover, future network states are generally unavailable when the current scheduling decision is made. Therefore, we transform the long-term delay minimization objective into a per-slot forwarding-efficiency maximization problem. The basic rationale is that selecting relay links with larger positive forwarding progress and sufficient link quality in each slot can reduce the expected number of remaining hops, thereby contributing to lower end-to-end delay. This transformation enables online scheduling based only on the currently observed network state, which is more suitable for highly dynamic mmWave V2V networks.

## 4. Proposed Scheme

The proposed IARS scheme is designed according to the structure of the evolved per-slot optimization problem. Since the problem involves binary link-selection variables, IRS resource constraints, half-duplex node constraints, and nonlinear interference constraints, solving it optimally in each time slot would incur high computational complexity. To make the problem tractable, we decompose it into two coupled subproblems: IRS resource allocation and direct relay selection. IRS-assisted links are considered first because IRS resources are scarce and reflection links can significantly improve NLOS transmission opportunities when properly configured. Therefore, we allocate IRS resources according to the relative performance gain of reflection links over their best direct-link alternatives. After IRS-assisted transmissions are determined, the remaining direct relay selection problem is modeled as a conflict graph, where each vertex represents a candidate relay link and each edge represents a node-sharing or interference conflict. In this way, selecting non-conflicting relay links is equivalent to finding a weighted independent set. Since the maximum weighted independent set problem is NP-hard, we adopt a greedy approximation based on the link weight and conflict degree, which provides a practical tradeoff between forwarding efficiency and per-slot computational complexity. The derivations and proofs of the key equations and algorithmic properties are provided in Appendix A.

### 4.1. Relay Link Evaluation Function

A vehicle node that can establish a link with the current transmitter $v_{i}$ through mmWave channels is one of the neighbors of $v_{i}$. Each neighbor of $v_{i}$ may be selected as a relay node to forward data packets. Thus, it is called a candidate relay node. A relay-link evaluation function is needed to determine the forwarding priority of each link associated with candidate relay nodes. The relay-link evaluation function provides a basis for determining whether a link should be selected for forwarding and whether IRS resources should be allocated to it.

The relay link evaluation function needs to meet several requirements. First, it should evaluate link effectiveness. The farther the relay node is from the current node and the closer the relay node is to the destination, the faster the data packet can be forwarded to the destination, which reduces the total multi-hop transmission delay. Therefore, the single-hop distance is an important metric for evaluating relay links. Due to the grid-like topology of urban streets, Manhattan distance more accurately reflects packet-forwarding distance than Euclidean distance. Let $Dis_{i}$ represent the Manhattan distance from the current node $v_{i}$ to the destination, and $Dis_{j}$ represent the Manhattan distance from $v_{j}$ to the destination, then $Dis_{i,j}=Dis_{i}-Dis_{j}$ represents the distance that the data packet is forwarded from $v_{i}$ to $v_{j}$.

Second, link quality is also a factor to consider. Although selecting a farther relay node can increase packet-forwarding distance and reduce multi-hop delay, atmospheric absorption degrades link quality over longer distances and increases the probability of transmission failure. Therefore, when selecting a relay node, it is often necessary to balance the distance and link quality. Before completing the scheduling decision, the interference is uncertain. In fact, one objective of scheduling is to avoid interference conflicts. Therefore, we adopt the rate ${\bar{R}}_{i,j,s}$ calculated by the signal-to-noise ratio ${SNR}_{i,j,s}$ of the link to reflect the link quality without interference.

According to the above, the relay link evaluation function for the reflective link of IRSs (s≠0) or the direct link (s=0) between node $v_{i}$ and candidate relay node $v_{j}$ is as follows:

(13) $$f\left(i,j,s\right)=\frac{Dis_{i}-Dis_{j}}{Dis_{max}}\frac{{\bar{R}}_{i,j,s}}{{\bar{R}}_{max}}$$

where $Dis_{max}$ and ${\bar{R}}_{max}$ are the farthest transmission distance and the maximum data rate of a single hop, respectively. The relay evaluation function in (13) is designed to balance forwarding progress and transmission reliability. The normalized distance term encourages the selection of relay vehicles that can provide larger progress toward the destination, which helps reduce the number of relay hops. In contrast, the normalized rate term reflects the achievable transmission quality of the candidate link and prevents the selection of long-distance but unreliable links. The multiplicative weighted formulation is adopted because it jointly penalizes poor performance in either term; that is, a candidate relay can obtain a high score only when it provides both sufficient forwarding progress and reliable link quality. The weighting parameter controls the relative preference between distance-oriented forwarding and rate-oriented reliable transmission. A detailed sensitivity analysis of this weighting formulation is provided in Appendix A.

### 4.2. IRS Allocation Scheme

The path loss of IRS-assisted communication has been characterized through experimental validation and communication-oriented modeling [23,24]. In a single-user case, where the IRS assists only one reflection link between a transmitter and a receiver, the results of related works and the path loss formulation for IRS-assisted communication show that the maximum reflection gain provided by the IRS is closely related to the size of the reflective surface (or the number of reflective elements). However, the situation is more complicated when the IRS assists multiple reflection links at the same time. An IRS configuration can only provide high gains for signals with specific incident angles and reflection angles. An IRS configuration that is optimal for one transmission may not be optimal for other transmissions. Thus, it is difficult to choose a configuration that is optimal for all transmissions. Existing works typically formulate IRS configuration for multiple assisted links as a network utility maximization problem, that is, maximizing the overall link quality or transmission utility while ensuring that all assisted transmissions satisfy minimum link-quality or quality of service (QoS) constraints [21,24]. Compared with the maximum gain that can be obtained when one link monopolizes the IRS, in this case, the reflection gain obtained by each link is reduced. If there are too many reflection links assisted by an IRS at the same time, the quality of some of the reflection links may not be as good as that of the direct link, or even fail to meet the minimum link quality requirements. Therefore, the number of reflection links that an IRS can simultaneously assist is limited. Different from IRS reflection-coefficient optimization for a given set of communication links, the IRS allocation problem considered in this paper determines which transmitters should obtain IRS assistance before relay scheduling. This is necessary because multiple reflection links may compete for the same IRS, while an IRS configuration can only provide sufficient gain for a limited number of incident-reflection angle pairs. Therefore, IRS allocation is treated as a competitive resource allocation problem jointly coupled with relay selection and interference-aware scheduling.

To reasonably allocate IRS resources in IRS-assisted mmWave vehicular networks, we design an IRS allocation scheme that addresses the following two problems. The first problem is how reflected links should compete for IRS resources when such resources are scarce relative to transmission demands, i.e., which links should be served preferentially. The second problem is determining when IRS resource allocation should be terminated. In other words, how to judge that an IRS has reached full capacity and can no longer serve any more links.

Before presenting the IRS allocation algorithm, we first evaluate the performance improvement obtained by a link after receiving IRS resources, which serves as its competition priority. The relay link evaluation function proposed in the previous subsection is used here. We set the priority of link $l_{i,j,s}$ to obtain IRSs assistance as follows:

(14) $$pri_{i,j,s}=\frac{f_{0}\left(i,j,s\right)}{\text{max}\left(\varepsilon ,\underset{k}{\text{max}}f\left(i,k,0\right)\right)}-1$$

where ε is a small positive number used to handle non-positive denominators and $f_{0}\left(i,j,s\right)$ represents the evaluation function under the maximum reflection gain, where the reflection link $l_{i,j,s}$ exclusively occupies IRSs. The priority represents the performance improvement rate of each reflection link compared to the direct link after obtaining the same IRS resource. Therefore, the higher the priority of a reflection link, the greater the benefit of allocating IRS resources for the transmitter. If the priority is negative, it implies that the reflection link is not as good as the direct link. After calculating the priority of each reflected link, IRS resource allocation can be performed accordingly.

Algorithm 1 shows the proposed IRS resource allocation algorithm. For each packet *d* on the network that has not yet reached the destination, we select the vehicle $v_{i}$ that holds the packet *d* and adopt it as a transmitter. $V_{t}$ denotes the set of transmitters. Initially, for each transmitter $v_{i}$, we add every reflection link that $v_{i}$ can construct with a priority greater than 0 to a queue *q* sorted in descending priority order. Then, in each iteration, we remove the reflected link $l_{i,j,s}$ with the highest priority from queue *q* and determine whether the resource allocation process for IRSs should be terminated. In order to determine the load capacity of IRSs, we tentatively add link $l_{i,j,s}$ to the set of links assisted by IRSs, $L_{s}$. After adding $l_{i,j,s}$, we complete the initial configuration of IRSs and determine whether the received power at all receivers reaches the required threshold. To determine whether an IRS can serve all transmissions assigned to it, we optimize the configuration with a goal of maximizing the minimum received power in the initial configuration process. Specifically, for each tile in turn, we select the configuration that maximizes the minimum received power of all receivers. After adding $l_{i,j,s}$ and completing the initial configuration, if the minimum power received by all receivers is lower than the threshold $p^{r}$, resource allocation for IRSs should be stopped immediately. Specifically, $l_{i,j,s}$ should not be added to $L_{s}$ and all links adopting IRSs should be removed from queue *q*. On the other hand, if the addition of $l_{i,j,s}$ does not exceed the capacity of IRSs, resource allocation for IRSs should continue.

In order to avoid transmission failures caused by signal interference, it is necessary to check whether there is a signal conflict between the newly added link $l_{i,j,s}$ and the already selected links. Specifically, if there is a previously selected link $l_{x,y,x}$ that satisfies $\frac{p_{i,j,s}}{\sum_{s_{0}}p_{x,j,s_{0}}+N_{0}B}<SINR_{0}$ or $\frac{p_{x,y,z}}{\sum_{s_{0}}p_{i,y,s_{0}}+N_{0}B}<SINR_{0}$, $l_{i,j,s}$ conflicts with $l_{x,y,z}$, and $l_{i,j,s}$ cannot be selected. If link $l_{i,j,s}$ does not conflict with the existing links, we should update the set of reflection links assisted by IRSs and the set of remaining transmitters. Then, we select a packet *d* from the packets held by $v_{i}$ and set $\alpha_{i,j,s,d}=1$. After $l_{i,j,s}$ is selected, for any link $l_{x,y,z}$ in *q*, if x∈{i,j} or y∈{i,j}, we should delete link $l_{x,y,z}$ from *q* to satisfy the requirement of constraint (9) that any vehicle can only act as one of a transmitter or receiver at the same time.

Finally, we repeat this process until queue *q* is empty, and then we complete the allocation of IRS resources. The detailed complexity derivation of Algorithm 1 is provided in Appendix A. The proof also shows that the queue size strictly decreases during the allocation process, which guarantees the finite termination of the IRS resource allocation algorithm.
**Algorithm 1:** The IRS resource allocation algorithm.input  : $V_{t}$: the set of transmitters; *S*: the set of IRSs;output: α: the IRS selection result; $\bar{V_{t}}$: the set of remaining transmitters;$q=Ø,\bar{V_{t}}=V_{t};$for $s_{0}$ in *S* do      $L_{s_{0}}=Ø;$endfor $v_{i},v_{j}$ in $V_{t}$ do      if $pri_{i,j,s}>0$ then            push $l_{i,j,s}$ into *q*;      endendwhile q≠Ø do      Select $l_{i,j,s}$ from *q* with the largest pri;      Delete $l_{i,j,s}$ from *q*, $L_{s}^{\prime }=L_{s}^{\prime }\{l_{i,j,s}\};$      Complete the initialized configuration optimization of IRSs based on $L_{s}^{\prime };$;      if $\min_{l_{x,y,z}\in L_{s}^{\prime }}p_{x,y,z}<p^{r}$ then            for $l_{x,y,z}$ in *q* do                  if z=s then                        Delete $l_{x,y,z}$ from *q*;                  end            end      end      else if $l_{i,j,s}$ has no signal conflict with the already selected links then            $L_{s}=L_{s}^{\prime };$            $\bar{V_{t}}=\bar{V_{t}}-\left\{v_{i}\right\}$;            Select *d* that satisfies $Q_{i,d}=1$, let $\alpha_{i,j,s,d}=1$;            for $l_{x,y,z}$ in *q* do                  if x∈{i,j} or y∈{i,j} then                        Delete $l_{x,y,z}$ from *q*;                  end            end      endend

### 4.3. Relay Selection and Scheduling Scheme

After IRS resource allocation is completed in the previous subsection, reflected links assigned with IRS resources are prioritized during relay selection. Therefore, we need to design an algorithm to select relay nodes for the remaining transmitters. These transmitters need to send data packets to relay nodes through direct links. We need to select as many high-priority relay links as possible while avoiding conflicts. Recent mmWave V2X scheduling studies explicitly model inter-link conflicts and solve combinatorial link activation problems to improve spatial reuse [6,11], and the relay selection algorithm proposed in this section follows the same conflict-graph scheduling spirit. We solve this relay selection problem by transforming it into a maximum weighted independent set problem, which preferentially selects relay links with better evaluation functions while avoiding signal collisions. The algorithm attempts to achieve the goal of selecting the optimal relay nodes in each round of scheduling to improve the forwarding efficiency.

In each round of scheduling, after completing the selection of reflection links, an RSU needs to select transmitters and receivers to transmit through direct links. First, based on the collected vehicle information, the RSU can estimate the direct link quality between a transmitter and its candidate relay nodes through the propagation loss model based on the distance and obstacle information between vehicles, and then the RSU can determine the connectivity between nodes and calculate the evaluation function of each candidate relay node.

Based on the connectivity between nodes and the evaluation function of relay links, we can construct a graph for relay node selection. Each vertex of the graph represents a relay selection, which consists of a transmitter and a candidate relay node, and the weight of the vertex is the evaluation function of the relay link. The edges of the graph represent conflicts between two relay selections, either because the two links share the same node or because signal interference exists between them. An independent set of vertices in such a graph can represent a non-conflicting selection of relay links. Therefore, solving the maximum weighted independent set problem in this graph (which we call the relay selection graph) can complete the relay node selection for the remaining transmitters.

Algorithm 2 shows how to select relay nodes based on the relay selection graph. First, it is necessary to construct a relay selection graph G=(A,E), where *A* and *E* are the set of vertices and the set of edges, respectively. Each vertex $a_{i,j}$ in the graph *G* corresponds to a relay link $l_{i,j,0}$ and the evaluation function f(i,j,0) is the weight of the vertex. After Algorithm 1 is completed, the set $\bar{V_{t}}$ contains transmitters that have not been allocated with IRS resources and need to forward through direct links. Initially, for each transmitter $v_{i}$ in $\bar{V_{t}}$, we obtain all candidate relay links that do not conflict with IRS-assisted relay forwarding and whose evaluation function is greater than 0. Then, these transmitter and receiver combinations are added to the vertex set *A*. If two relay links conflict with each other and cannot be selected at the same time, then we add an edge between their corresponding vertices. Similar to Algorithm 1, relay-selection conflicts arise in two cases: when two links use the same node or when signal interference exists between links. After graph *G* is constructed, the relay node selection can be completed by solving its maximum weighted independent set problem. Since the maximum weighted independent set problem is NP-hard, we approximate it with a greedy algorithm. The algorithm sequentially selects the vertex with the largest f/(deg+1) from graph *G*, where *f* is the weight of the vertex, as well as the evaluation function of the corresponding relay selection, and deg is the degree of the vertex. After a vertex is selected, this vertex and its adjacent vertices should be removed from graph *G*. This process continues until there are no vertices in graph *G*. Thus, an independent set of vertices is selected, and this set of vertices corresponds to our relay selection. Therefore, for each selected vertex $a_{i,j}$, we select a packet *d* from those held by $v_{i}$ and set $\alpha_{i,j,0,d}=1$.

**Figure 5:**
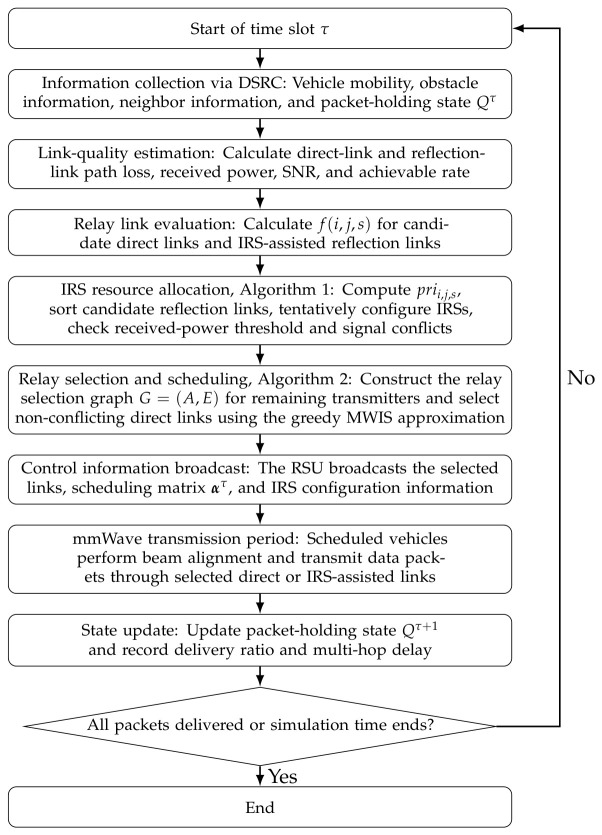
Flowchart of the proposed IARS method.

The above two algorithms are executed once in each scheduling time slot. Since both algorithms are deterministic greedy procedures, they terminate in a finite number of steps. In Algorithm 1, the candidate IRS-assisted link queue strictly decreases in each iteration, and thus the number of iterations is upper-bounded by the number of candidate reflected links. In Algorithm 2, at least one vertex is removed from the relay selection graph in each iteration, and thus the number of iterations is upper-bounded by the number of candidate direct relay links. Therefore, the proposed IARS scheme can always produce a scheduling decision after a finite number of operations in each time slot. Since the scheduling computation is performed by the RSU during the control period, which can overlap with the transmission period of the previous time slot, the finite-step property and polynomial complexity make the proposed scheme feasible for per-slot scheduling. The detailed complexity derivation and termination proof of Algorithm 2 are provided in Appendix A. Since at least one vertex is removed from the relay selection graph in each iteration, the greedy relay selection process is guaranteed to terminate within a finite number of steps. To further clarify the implementation procedure of the proposed IARS scheme and facilitate result reproduction, Figure 5 presents the overall workflow of the proposed method in each scheduling time slot.
**Algorithm 2:** The relay selection algorithm.input  : $\bar{V_{t}}$: remaining transmitters;output: α: the relay selection resultA=Ø,E=Ø;for $v_{i},v_{j}$ in $V_{t}$ do      if f(i,j,0)>0 and $l_{i,j,0}$ does not conflict with the already selected IRS-assisted links        then            $A=A\cup \{a_{i,j}\}$;      endendfor $a_{i,j},a_{x,y}$ in *A* do      if $a_{i,j}$ conflicts with $a_{x,y}$ then            $E=E\cup \{e_{a_{i,j},a_{x,y}}\}$;      endendwhile A≠Ø do      Select $a_{i,j}$ from *A* with the largest f/(deg+1);      Select *d* that satisfies $Q_{i,d}=1$, let $\alpha_{i,j,0,d}=1$;      Delete $a_{i,j}$ and its adjacent vertices from *A*;end

## 5. Performance Evaluation

**Figure 6:**
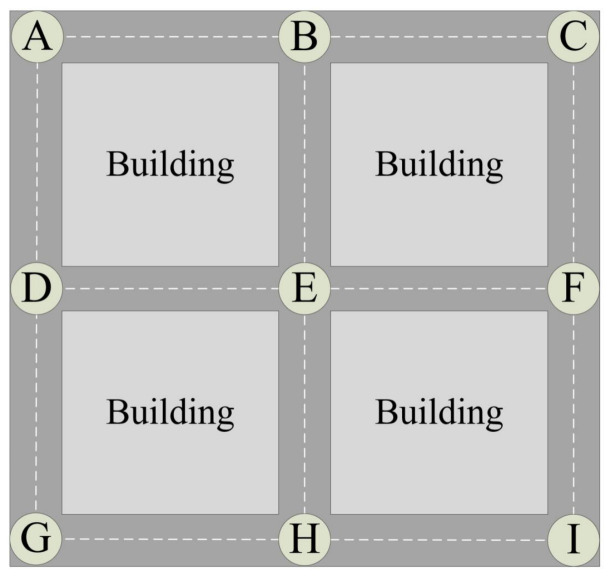
Streets layout in simulation. The letters mark the intersection.

In this section, we conduct simulation experiments using the OMNeT++ [25], Veins [26], and SUMO [27] frameworks. Since the physical and data-link layers of Veins are implemented based on the IEEE 802.11p [28] standard, we extend its physical layer to support mmWave antennas and implement an IRS module according to the transmission-mode-based IRS configuration method in [24]. We use SUMO to set up a street scene with a 2×2 grid structure, as shown in Figure 6. In this street scene, three roads in the horizontal direction and three roads in the vertical direction intersect at nine intersections A∼I, and intelligent reflecting surfaces are deployed at these nine intersections. Roads are divided into street segments by intersections, and each street segment is 200 m long. Each road has four 4 m wide lanes, two in each direction. Considering the existence of sidewalks in the urban scene, we set the total width of the street to 30 m. In other words, the obstacles on both sides of the street are 30 m apart. Traffic lights are set at intersections with a period of 20 s for red, 20 s for green and 3 s for yellow. Table 2 lists the detailed configuration parameters. All simulations were conducted on a workstation running Ubuntu 18.04 LTS. The hardware platform was equipped with an Intel Core i9-9900X CPU @ 3.50 GHz (Intel Corporation, Santa Clara, CA, USA) and 128 GB of memory. The simulation framework was implemented based on OMNeT++ 5.6.2, Veins 5.2, and SUMO 1.8.0. These configurations were used throughout the simulation experiments to ensure consistent and reproducible evaluation results.

To verify the necessity and effectiveness of deploying IRS to assist mmWave multi-hop V2V communication, we implement the proposed IARS scheme with and without IRS assistance. If the IARS scheme is adopted in a network where IRS is not deployed, the IRS allocation process is omitted, and relay node selection is directly performed. As discussed in Section 2, most recent mmWave/RIS-assisted vehicular communication schemes focus on beam alignment, beamforming, channel estimation, reflection coefficient optimization, single-link reliability, or two-hop cooperative transmission. They are not directly comparable with the packet-level multi-hop IRS allocation and relay selection problem considered in this paper. Therefore, instead of introducing an adapted but potentially unfair implementation of these methods, we adopt maximum distance forwarding (MDF) as a representative and reproducible distance-driven forwarding baseline. MDF is a widely used greedy forwarding principle for multi-hop communications, where each transmitter selects the candidate node closest to the destination as the next hop. In our implementation, MDF uses the same mobility traces, packet traffic, candidate link generation, IRS deployment, and SINR threshold as the proposed IARS scheme, but it does not consider the proposed transmission evaluation function, IRS resource competition, or concurrent interference-aware scheduling. Therefore, the comparison with MDF can isolate the contribution of the proposed IRS allocation and relay selection mechanism.

Specifically, we implement mmWave multi-hop V2V communication based on MDF in networks with and without IRS, respectively. In the MDF scheme, each transmitter selects the node closest to the destination as the next hop among the candidate nodes whose estimated SNR is higher than a threshold. Selecting the node closest to the destination helps reduce the overall multi-hop delay, but reliability may be degraded by interference from other transmitters. Compared with directly adopting DRL-based relay selection or existing IRS-assisted relay schemes, this controlled comparison avoids additional factors such as training convergence, reward design, reflection-coefficient optimization, and different single-/two-hop assumptions, and thus more clearly reveals the effects of IRS deployment and interference-aware relay selection. To sum up, we implement the following four schemes for mmWave multi-hop V2V communication:

- IARS with IRS: Adopting the proposed IARS scheme in a network with IRS.
- MDF with IRS: Adopting the maximum distance forwarding scheme in a network with IRS.
- IARS without IRS: Adopting the proposed IARS scheme in a network without IRS.
- MDF without IRS: Adopting the maximum distance forwarding scheme in a network without IRS.

**Figure 7:**
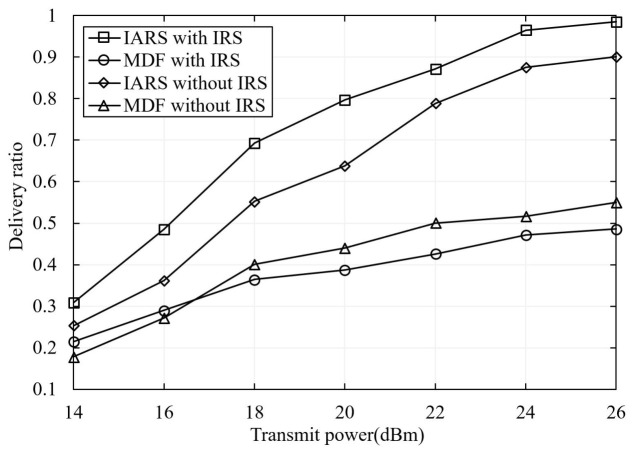
The delivery ratio for varying transmit powers.

Figure 7 plots the delivery ratio versus the transmitter power for four different schemes. The number of tiles configured for each IRS is 8 by default, and the average distance between adjacent vehicles is 60 m by default. It can be observed that in the case of low transmit power, the delivery ratio of multi-hop communication of all schemes is very low. Compared to the random distance between vehicles, the single-hop transmission distance of mmWave signals is limited when the transmission power is low. There may be no valid candidate relay node within the single-hop communication range of a transmitter; thus, forwarding at low transmit power suffers from a high outage probability. In addition, traffic lights may lead to uneven vehicle distributions on streets, increasing the probability of street disconnection and forwarding failures. As the transmit power increases, the delivery ratio of all schemes increases. This is because increasing transmit power improves the single-hop transmission distance, thereby reducing the probability of forwarding outages. On the other hand, higher transmit power can also improve the SINR of the signal received by the receiver and reduce the bit error rate. After adopting our proposed scheme, the delivery ratio is significantly improved both with and without the IRS. Our proposed scheme considers the influence of interference when selecting links, which avoids the occurrence of signal collisions and can significantly improve the transmission success ratio. In addition, IRS deployment can further improve the delivery ratio when the IARS scheme is adopted. This is because reflection links can increase the single-hop transmission distance and bypass obstacles, thereby establishing connections with more nodes and reducing the probability of multi-hop outage. If IRS were deployed in a network without adopting the IARS scheme, the increase in the delivery ratio is not significant compared to the case without IRS. This is because the occurrence of reflection signals makes the signal interference more serious after the IRS is deployed. If the interference is not considered when selecting links, signal collisions may seriously affect the transmission success ratio.

**Figure 8:**
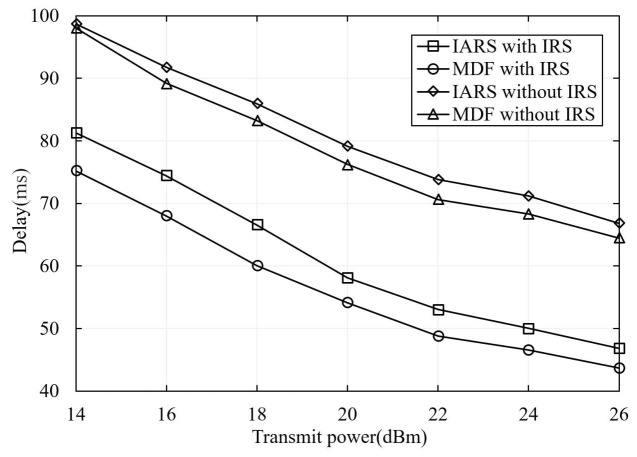
The multi-hop delay for varying transmit powers.

Figure 8 shows the multi-hop delay versus the transmitter power for four different schemes. It can be observed that, for all schemes, increasing transmit power extends the single-hop transmission distance and thereby reduces multi-hop delay. In addition, deploying IRS can significantly improve the multi-hop delay performance, which is mainly achieved by increasing the single-hop transmission distance and reducing the number of hops. On the other hand, the IARS scheme proposed in this paper achieves slightly lower delay performance than the maximum-distance forwarding scheme. This is mainly because some nodes with the largest forwarding distance are not selected during link selection in order to avoid signal interference. However, this latency tradeoff is acceptable considering the resulting reliability improvement.

**Figure 9:**
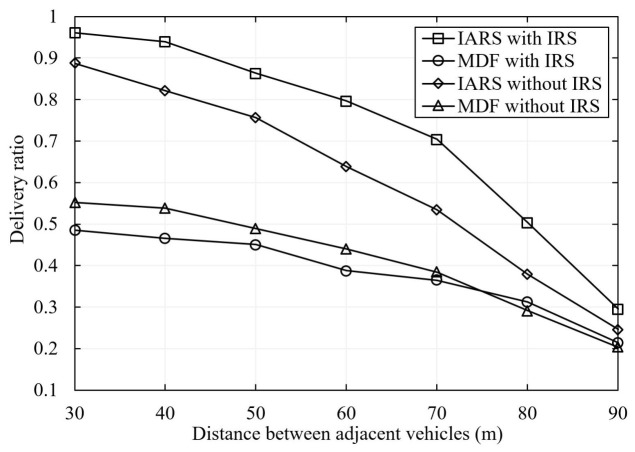
The delivery ratio for varying distances between adjacent vehicles.

Figure 9 illustrates the delivery ratio versus the distance between adjacent vehicles in a lane for four different schemes. As the distance between adjacent vehicles increases, the effective candidate relay nodes within the transmitter’s single-hop range decrease, thereby increasing the outage probability of multi-hop communication. It can be observed that adopting the IARS scheme in a network with IRS can achieve the highest delivery ratio. This is because deploying IRS improves the single-hop transmission range, and the IARS scheme considers the influence of interference in the selection of links, reducing the signal collision probability.

**Figure 10:**
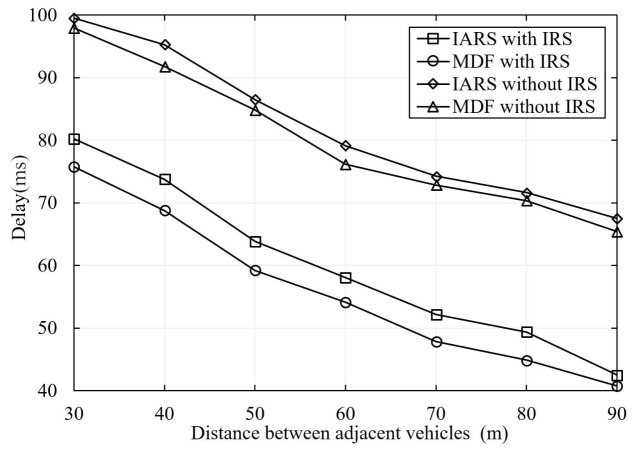
The multi-hop delay for varying distances between adjacent vehicles.

Figure 10 shows the relationship between the multi-hop delay and the distance between adjacent vehicles in a lane for four different schemes. As the distance between adjacent vehicles increases, the multi-hop delay significantly decreases. If the distance between vehicles is small and the density is high, the transmission is easily affected by vehicle obstacles, which results in limited single-hop transmission distance and long multi-hop delays. Reflection links constructed by the IRS can bypass obstacles, reduce the number of hops, and improve the delay performance.

**Figure 11:**
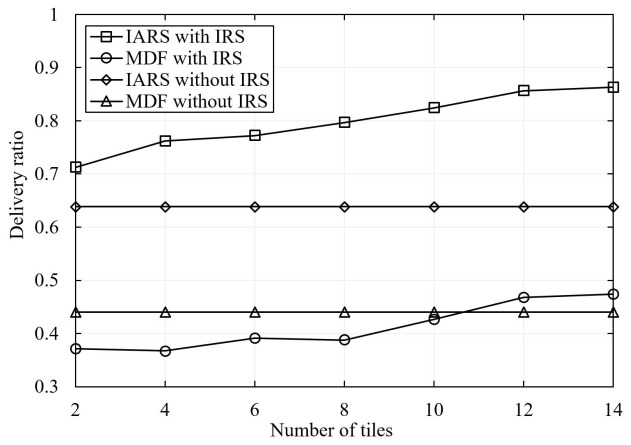
The delivery ratio for varying the number of tiles.

**Figure 12:**
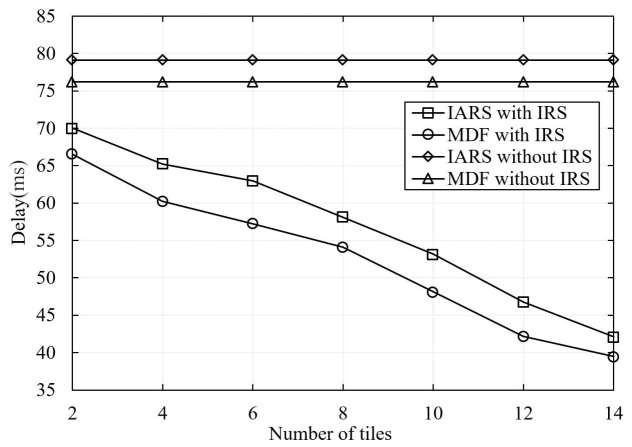
The multi-hop delay for varying the number of tiles.

Figure 11 and Figure 12 plot the delivery ratio and multi-hop delay, respectively, versus the number of tiles configured for an IRS. It can be observed that, as the number of tiles configured for an IRS increases, more reflective resources become available, leading to greater performance gains from IRS assistance. The reliability and delay performance of multi-hop transmissions can be significantly improved with the increase in the number of tiles configured for an IRS.

To further distinguish the contribution of IRS allocation from that of relay selection and scheduling, we conduct an ablation study under the default setting, where the transmit power is 20 dBm, the average inter-vehicle distance is 60 m, and each IRS is configured with eight tiles. The results are summarized in Table 3. All results are obtained from 100 independent simulation runs with 95% confidence intervals.

By comparing IARS with IRS and IARS without IRS, it can be observed that removing IRS decreases the delivery ratio from 0.80 to 0.63, corresponding to a 21.2% reduction with respect to the complete scheme. Meanwhile, the average delay increases from 59 ms to 79 ms, corresponding to a 33.9% increase. This result confirms that IRS-assisted reflection links can effectively improve link availability, bypass urban blockage, and reduce the number of hops in mmWave multi-hop V2V communication. By comparing IARS with IRS and MDF with IRS, the delivery ratio decreases from 0.80 to 0.39 when the proposed relay selection and scheduling algorithm is replaced by MDF, corresponding to a 51.2% reduction. This demonstrates that IRS deployment alone is insufficient if the interference among direct and reflected links is not properly handled. Although MDF with IRS achieves a slightly lower delay than IARS with IRS, this is mainly because MDF tends to select longer forwarding links among successfully delivered packets. However, such distance-oriented forwarding ignores inter-link interference and therefore suffers from a much lower delivery ratio. In addition, the delivery ratio of MDF with IRS is even slightly lower than that of MDF without IRS, which further indicates that unmanaged IRS reflections may introduce additional interference when IRS allocation and interference-aware scheduling are not jointly considered. Therefore, the proposed IARS scheme benefits from both IRS-assisted link enhancement and interference-aware relay scheduling, with the latter being essential for converting IRS resources into reliable end-to-end performance gains.

To further evaluate the robustness and scalability of the proposed scheme, we extend the simulation scope by considering different grid sizes and vehicle densities. Specifically, in addition to the default 2×2 grid, we further construct a larger 3×3 urban grid in SUMO, which contains more intersections, longer potential multi-hop paths, and more possible link conflicts. Meanwhile, three vehicle densities, i.e., 10, 17, and 40 veh/km/lane, are tested to represent sparse, default, and dense traffic conditions, respectively. The results are summarized in Table 4. It can be observed that the proposed IARS with IRS consistently achieves the highest delivery ratio under all tested grid sizes and vehicle densities. When the grid size increases from 2×2 to 3×3, the delivery ratio of all schemes decreases and the multi-hop delay increases, since packets need to traverse longer paths and the number of potential transmission conflicts becomes larger. Nevertheless, IARS with IRS still maintains clear reliability advantages. For example, in the 3×3 grid, IARS with IRS achieves delivery ratios of 0.30, 0.70, and 0.81 under 10, 17, and 40 veh/km/lane, respectively, while MDF with IRS only achieves 0.14, 0.29, and 0.36. This confirms that the proposed interference-aware relay selection and IRS allocation remain effective when the network scale expands.

The results also show the impact of traffic density. Under sparse traffic, the delivery ratio is relatively low because the number of available relay candidates is limited. As the vehicle density increases, more relay candidates become available, thereby improving network connectivity and delivery ratio. However, dense traffic also introduces more blockage and concurrent transmission conflicts, leading to longer multi-hop delay. Compared with IARS without IRS, IARS with IRS significantly reduces the delay in both grid sizes, because IRS-assisted reflection links can bypass blocked road segments and reduce the number of forwarding hops. Although IARS may introduce slightly higher delay than MDF due to its interference-aware link selection, this delay cost is acceptable considering the substantial improvement in delivery reliability.

## 6. Conclusions

We proposed deploying IRSs on urban streets to assist mmWave multi-hop V2V communication. Directional mmWave signals allow a transmitter to select either a direct link or a reflective link for transmission. However, the number of transmissions that an IRS can assist simultaneously is limited; thus, an IRS allocation scheme is required. In addition, after the introduction of IRS, reflection signals may exacerbate the transmission interference between vehicles. If IRSs are deployed without effective measures to avoid signal collisions, the reliability of V2V communication may be seriously degraded. To fully exploit the transmission-performance gains provided by reflected links and improve the reliability and delay of mmWave multi-hop V2V communication, we proposed an efficient IRS allocation and relay selection scheme, called IARS. Taking into account the load capacity of each IRS, the IARS scheme selects transmitters that need to transmit through reflection links most urgently and allocates IRS resources to them. Then, the IARS scheme jointly considers forwarding distance, link quality, and concurrent conflicts to perform relay selection. Simulation results show that the proposed IARS scheme effectively improves reliability and reduces the multi-hop delay of mmWave multi-hop V2V communication in IRS-assisted vehicular networks. In addition, the comparison with MDF is intended to reveal the effect of interference-aware IRS allocation and relay selection under the same packet-level simulation environment, while direct benchmarking against recent PHY-layer RIS/beamforming methods will be considered in future work when their assumptions and control variables can be fairly unified with multi-hop vehicular forwarding.

It is worth noting that the present work focuses on a lightweight and interpretable design, where IRS allocation and relay selection are performed according to link quality, forwarding progress, and concurrent interference. More advanced learning-based methods, such as DRL-enabled relay selection and joint IRS/relay coordination, may further improve the adaptability of the system under highly dynamic traffic conditions. However, such methods require dedicated state representation, action-space design, reward shaping, and training under IRS-assisted multi-hop V2V scenarios. We will investigate DRL-based IRS allocation and relay selection as an important direction in future work.

## Figures and Tables

**Table 1:** Summary of key related work.

Category	Representative Works	Pros	Cons/Gaps	Our Solution
mmWave V2X scheduling	[5,6,11,15]	Improve beam alignment, scheduling efficiency, and link adaptability in mmWave V2X networks	Mainly focus on link-level scheduling or beam management, without considering IRS-assisted multi-hop relay forwarding	We jointly consider direct and IRS-assisted links for multi-hop V2V relay selection
Vehicular blockage and relay transmission	[10,16,17]	Reveal the severe impact of vehicular blockage and show the benefit of relay-assisted transmission	Do not fully exploit IRS reflection to overcome NLOS blockage and expand relay opportunities	We use IRS-assisted reflected links to improve connectivity under blockage
IRS/RIS-enabled vehicular communication	[8,14,18,19]	Provide useful IRS/RIS channel models, beamforming methods, and vehicular communication frameworks	Mostly focus on channel modeling or single-link enhancement, rather than end-to-end multi-hop forwarding	We integrate IRS assistance into the multi-hop routing and scheduling process
RIS/relay cooperative transmission	[12,20]	Analyze the reliability gain of RIS/relay cooperation and interference-aware transmission	Usually treat RIS and relay as separate or alternative mechanisms, with limited attention to IRS resource competition	We jointly optimize IRS allocation, relay selection, and concurrent transmission scheduling
IRS-assisted multi-hop vehicular communication	[13]	Closely related to IRS-assisted multi-hop vehicular communication	Does not sufficiently consider limited IRS resources, directional interference, and concurrent relay scheduling together	We design an IRS allocation and relay scheduling scheme considering link quality, forwarding progress, and interference

**Table 2:** Simulation Parameters.

SUMO Simulation Setup	
Default vehicle speed	50 km/h
Default distance between adjacent vehicles	60 m
Number of lanes	4 (2 per direction)
Lane width	4 m
Street width	30 m
Street segment length	200 m
Traffic light cycle	(R, Y, G) = (20, 3, 20) s
Car following model	Krauss model
Lane change model	LC2013
General Simulation Parameters	
Frequency band	60 GHz
Bandwidth *B*	1.08 GHz
Noise power density $N_{0}$	−174 dBm/Hz
SINR threshold	5 dB
half power beamwidth $\phi^{t},\phi^{r}$	$10^{\circ }$
Number of elements in a tile	100
Data packet generation rate	200/s
Length of data packet	1 Mbits
Default number of tiles	8
Default transmit power	20 dBm
Simulation time	200.2 s
Simulation times	100
Confidence level	95%

**Table 3:** Ablation Study Results under the Default Setting.

Scheme	Delivery Ratio	Delay (ms)	Note
IARS with IRS	0.80	59	Complete scheme
IARS without IRS	0.63	79	Without IRS assistance
MDF with IRS	0.39	54	Without IARS scheduling
MDF without IRS	0.44	76	Without IRS and IARS scheduling

**Table 4:** Scalability Evaluation: Delivery Ratio and Delay under Different Grid Sizes and Vehicle Densities.

Grid Size	Vehicle Density (veh/km/lane)	IARS w/IRS	MDF w/IRS	IARS w/o IRS	MDF w/o IRS
Delivery Ratio					
	10	0.30 ± 0.003	0.16 ± 0.002	0.26 ± 0.003	0.19 ± 0.003
2 × 2	17	0.80 ± 0.006	0.39 ± 0.003	0.64 ± 0.005	0.45 ± 0.005
	40	0.95 ± 0.011	0.48 ± 0.004	0.88 ± 0.009	0.56 ± 0.005
	10	0.30 ± 0.003	0.14 ± 0.002	0.23 ± 0.002	0.17 ± 0.001
3 × 3	17	0.70 ± 0.011	0.29 ± 0.004	0.53 ± 0.005	0.34 ± 0.003
	40	0.81 ± 0.008	0.36 ± 0.004	0.70 ± 0.008	0.42 ± 0.006
Multi-hop Delay (ms)					
	10	43 ± 0.4	40 ± 0.5	66 ± 1.0	63 ± 0.7
2 × 2	17	58 ± 0.8	54 ± 0.8	79 ± 0.6	76 ± 0.8
	40	80 ± 1.0	76 ± 0.6	98 ± 0.9	95 ± 1.1
	10	66 ± 1.0	61 ± 0.5	99 ± 1.4	96 ± 0.8
3 × 3	17	88 ± 0.8	83 ± 1.2	123 ± 1.3	119 ± 1.2
	40	116 ± 1.0	113 ± 1.0	153 ± 2.2	150 ± 1.2

## Data Availability

The raw data supporting the conclusions of this article will be made available by the authors on request.

## Appendix A. Complexity, Convergence, and Sensitivity Analysis

This appendix provides the derivations and proofs related to the key equations and algorithmic properties of the proposed IARS scheme. We first derive the computational complexity of the IRS allocation algorithm and the relay selection algorithm. Then, we prove their finite termination and discuss the feasibility of executing the proposed scheduling process within each time slot. Finally, we analyze the sensitivity of the relay link evaluation function in Equation (13).

### Appendix A.1. Notations

Let $N_{v}$ denote the number of vehicles, $N_{t}$ denote the number of transmitters in one time slot, *S* denote the number of IRSs, and *C* denote the maximum number of candidate relay nodes for each transmitter. Then, the number of candidate reflected links is upper-bounded by

(A1) $$K=O(N_{t}CS).$$

Let *N* and *M* denote the number of IRS tiles and the number of optional transmission modes for each tile, respectively.

### Appendix A.2. Complexity of Algorithm 1 (IRS Allocation)

Algorithm 1 first constructs a priority queue for candidate IRS-assisted reflected links. Since each transmitter has at most *C* candidate receivers and each pair may use at most *S* IRSs, the number of candidate reflected links is $K=O(N_{t}CS)$. Building and sorting the queue requires

(A2) O(K log K)

time.

In each iteration, the algorithm selects the highest-priority reflected link, tentatively adds it to the corresponding IRS-assisted link set, and performs the initialized IRS configuration optimization. For each IRS, there are *N* tiles and *M* modes per tile, and the received power of all assigned links must be evaluated. Thus, one configuration step has complexity

(A3) $$O(NMN_{t}).$$

The while-loop executes at most *K* times. Additionally, deleting conflicting links or links involving the same transmitter/receiver requires scanning the queue, costing O(K) per iteration. Therefore, the worst-case complexity of Algorithm 1 is

(A4) $$O\left(K\;\text{log}\;K+K(NMN_{t}+K)\right).$$

Substituting $K=O(N_{t}CS)$ gives

(A5) $$O\left(N_{t}CS\;\text{log}\left(N_{t}CS\right)+N_{t}^{2}CSNM+N_{t}^{2}C^{2}S^{2}\right).$$

If C,S,N,M are bounded constants, the complexity reduces to quadratic in $N_{t}$:

(A6) $$O(N_{t}^{2}).$$

### Appendix A.3. Complexity of Algorithm 2 (Relay Selection)

Algorithm 2 constructs a relay selection graph. Each vertex corresponds to a candidate direct relay link, and the number of vertices is bounded by $P=O(N_{t}C)$. Constructing vertices costs O(P). Checking pairwise conflicts to build edges requires $O(P^{2})$.

The greedy selection iteratively chooses the vertex with largest f/(deg+1), removes it and its neighbors. Each iteration costs O(P), and at most *P* iterations occur. Therefore, the total complexity of Algorithm 2 is

(A7) $$O\left(P^{2}\right)=O\left(N_{t}^{2}C^{2}\right).$$

If *C* is bounded, the complexity simplifies to

(A8) $$O(N_{t}^{2}).$$

### Appendix A.4. Convergence and Time-Slot Feasibility

The proposed IARS scheme is designed for per-slot scheduling. Although the original optimization problem is nonlinear and combinatorial, the proposed scheme does not rely on an iterative nonlinear optimizer. Instead, it adopts two deterministic greedy algorithms. Therefore, the convergence of IARS refers to the finite termination of the scheduling procedure within each time slot.

For Algorithm 1, the priority queue contains at most $K=O(N_{t}CS)$ candidate IRS-assisted reflected links. In each iteration, one candidate link is selected and removed from the queue. In addition, some conflicting links or links associated with the same transmitter or receiver may also be removed. Thus, the size of the queue strictly decreases in every iteration, and Algorithm 1 terminates after at most *K* iterations.

For Algorithm 2, the relay selection graph contains at most $P=O(N_{t}C)$ vertices. In each iteration, one vertex is selected, and this vertex and its adjacent vertices are removed from the graph. Therefore, at least one vertex is removed in every iteration, and Algorithm 2 terminates after at most *P* iterations.

Accordingly, the proposed IARS scheme always converges to a feasible scheduling decision in a finite number of steps for each time slot. Combining the complexity analysis above, when the numbers of candidate relay nodes, IRSs, IRS tiles, and optional tile modes are bounded by practical deployment constraints, the overall computational complexity grows polynomially, and is quadratic with respect to the number of active transmitters.

In the considered time-slot structure, the RSU performs information collection, IRS allocation, relay selection, and control information broadcast during the control period. This control period can overlap with the data transmission period of the previous time slot. Therefore, the scheduling decision only needs to be completed before the corresponding transmission period starts. The finite-step convergence and polynomial complexity of the proposed algorithms ensure that the proposed IARS scheme is feasible for per-slot scheduling in the considered mmWave V2V network.

### Appendix A.5. Sensitivity Analysis of Equation (13)

The relay evaluation function in Equation (13) is

(A9) $$f\left(i,j,s\right)=\frac{Dis_{i}-Dis_{j}}{Dis_{\text{max}}}\frac{R_{i,j,s}}{R_{\text{max}}}.$$

The first term measures normalized forwarding progress, while the second term measures normalized link quality. This multiplicative form ensures that a candidate link is only highly ranked if it simultaneously makes sufficient progress and provides reliable transmission.

We can generalize Equation (13) with a weighting parameter ω:

(A10) $$f_{\omega }\left(i,j,s\right)={\left(\frac{Dis_{i}-Dis_{j}}{Dis_{\text{max}}}\right)}^{\omega }{\left(\frac{R_{i,j,s}}{R_{\text{max}}}\right)}^{1-\omega },\;0\leq \omega \leq 1.$$

When ω→1, the algorithm prioritizes forwarding distance; when ω→0, it prioritizes link quality. For two candidate links *a* and *b*, the critical value at which the ranking changes is

(A11) $$\omega^{*}=\frac{\text{ln}({\tilde{R}}_{b}/{\tilde{R}}_{a})}{\text{ln}\left({\tilde{D}}_{a}/{\tilde{D}}_{b}\right)+\text{ln}\left({\tilde{R}}_{b}/{\tilde{R}}_{a}\right)}.$$

Thus, small variations in ω generally do not change the selected relay unless two links are closely balanced. The default multiplicative choice ω=0.5 provides a reasonable trade-off between delay reduction and link reliability.
